# Recent advances in understanding the role of eosinophils

**DOI:** 10.12703/r/11-26

**Published:** 2022-09-27

**Authors:** Gregory M Constantine, Amy D Klion

**Affiliations:** 1 Human Eosinophil Section, National Institute of Allergy and Infectious Disease, National Institutes of Health, Bethesda, MD, USA

**Keywords:** eosinophils, eosinophil heterogeneity, eosinophil-targeted therapies, novel treatment strategies, host-viral defense

## Abstract

Our understanding of eosinophil biology, development, and regulation has dramatically increased in the past decade, leading to new paradigms for the role of eosinophils in human health and disease and, perhaps more importantly, providing insights toward novel treatment strategies in the fight against eosinophil-mediated inflammation. In this review, we discuss recent advances regarding the role of eosinophils in host-viral defense, eosinophil heterogeneity, and eosinophil-targeted therapies.

## Introduction

Conserved throughout evolutionary history, the eosinophil has long been recognized for its role in innate parasite immunity and type 2 inflammation^1,2^. Over the last decade, however, the role of eosinophils in a wide variety of homeostatic processes, including antitumor responses, tissue remodeling and fibrosis, metabolism, and immunoregulation, has become evident. This, combined with the development of novel therapeutic agents that target eosinophils^3^, has led to an increasing appreciation of the multifunctional nature of this enigmatic cell. In 2021, a total of 4,764 publications were identified in PubMed using the search term “eosinophil OR eosinophilia,” a 72% increase from 2,770 in 2011. In comparison, the number of publications retrieved using “mast cells OR mastocytosis” increased only 28.4% (from 1,423 to 1,827) during the same time frame. Given the breadth of recent information pertaining to eosinophil biology and function, a comprehensive summary is beyond the scope of this review. Instead, we will focus our discussion on three topics of considerable recent interest: (1) the antiviral response, including findings related to severe acute respiratory syndrome coronavirus-2 (SARS-CoV-2), (2) eosinophil development and heterogeneity, and (3) lessons learned from the rapidly evolving landscape of anti-eosinophil therapy.

## Eosinophils and the antiviral response

The association of eosinophils with viral infection was first postulated in 1950 by Robert Kaufman, who described mild eosinophilia in nearly 25% of patients with infectious mononucleosis^4^. Despite this early observation, the role of eosinophils in the immune response to viral infection was virtually unstudied until the 1969 description of pulmonary eosinophils in two children who died from a hypersensitivity reaction to the respiratory syncytial virus (RSV) following immunization with a formalin-inactivated RSV vaccine^5^. These initial findings and the association between early RSV infection and wheezing in childhood^6^ paved the way for studies exploring the role of eosinophils in viral infection (reviewed in 7).

### The eosinophil and respiratory viruses

Recruitment of eosinophils to the lung has been demonstrated in mice infected with a wide variety of respiratory viruses, including RSV and influenza virus. Moreover, several lines of evidence support the direct and indirect roles of eosinophils in the immune response to viral pathogens. Human and mouse eosinophils express a variety of surface receptors, including Toll-like receptors and retinoic acid-inducible gene I (RIG-I), that recognize viral nucleic acid motifs^8–10^. Detection of these motifs can cause eosinophil activation with the release of reactive oxygen species^11,12^, cytokines^13^, and eosinophil cationic granule proteins, several of which have potent ribonuclease activity^14^. Mouse and human eosinophils have also been shown to internalize and inactivate respiratory virus (influenza and RSV) particles in a process that leads to eosinophil activation and release of interleukin 6 (IL-6) and IL-8. Eosinophils indirectly modulate viral immune responses through their function as antigen-presenting cells, as evidenced by *in vitro* studies demonstrating antigen-specific activation of virus-specific T cells by eosinophils exposed to rhinovirus or influenza virus and by adoptive transfer experiments of lung eosinophils from fungal allergen-challenged mice into the tracheas of mice with influenza infection^15,16^. More recently, the same group showed that lung eosinophils rapidly upregulate the adhesion molecules, VLA-4 and ICAM-1, in murine influenza infection, migrate to the T-cell zones of the mediastinal lymph nodes, and protect the airway barrier from cytotoxic damage, possibly through downregulation of pro-apoptotic genes in infected epithelial cells^17^.

Viral respiratory infections remain a leading cause of asthma exacerbations and hospitalizations^18^. Whereas most data suggest impairment of innate immune mechanisms in asthmatics^19–22^, this appears to be counterbalanced by increased allergen-activated eosinophils in the lung, raising concern that therapeutic depletion of eosinophils might lead to increased frequency or severity of viral infection. This was explored directly in a placebo-controlled study of patients with mild asthma treated with the anti-IL-5 monoclonal antibody (mAb), mepolizumab, and challenged with rhinovirus^23^. Although mepolizumab treatment affected both the cellular and humoral responses to rhinovirus challenge and resulted in increased viral loads, eosinophil activation by rhinovirus was preserved and there was no effect of eosinophil depletion on clinical symptoms of viral infection. This lack of adverse outcomes is supported by the data from multiple clinical trials of eosinophil-depleting therapy in asthmatic patients, which have shown no increase in frequency or severity of viral infections but rather a reduction in acute asthma exacerbations^24–26^. Ultimately, continued investigation is needed to better understand eosinophil-viral interactions in the context of atopic disease.

### The SARS-CoV-2 pandemic

In December 2019, reports of a mysterious viral pneumonia emerged from Wuhan, China^27,28^. The pathogen would quickly be identified as the novel beta-coronavirus, SARS-CoV-2, and the infection as coronavirus disease 2019 (COVID-19)^29^. The rapid spread of the virus has led to an unprecedented health crisis and a global pandemic with over 167 million infections and more than 3.4 million deaths worldwide in the year following the initial report^30^. Although the association between eosinopenia and disease severity in patients with COVID-19^31–33^ has led some to suggest that eosinopenia might distinguish SARS-CoV-2 infection from other viral respiratory illnesses^34,35^, it is important to recognize that febrile eosinopenia was first recognized by Zappert^36^ in 1893, nearly 10 years prior to the description of the first human viral pathogen. Moreover, eosinopenia is a well-established diagnostic sign and prognostic indicator in critically ill patients with sepsis^37–42^ and was a marker of severe disease during the 2009 H1N1 influenza pandemic, affecting 50% of patients at the time of hospital admission^43^. The mechanism underlying febrile eosinopenia remains incompletely understood, although recent data implicate CD49d-mediated homing of eosinophils to tissues^44^ and exuberant type 1 immunity or type I interferon-mediated eosinophil apoptosis^45,46^.

The role of eosinophils in COVID-19-associated pathology continues to be controversial. Early studies suggested that eosinophilic pulmonary inflammation was uncommon in COVID-19^47,48^, and histopathological studies demonstrated a proinflammatory infiltrate comprised predominately of neutrophils, monocytes, macrophages^49,50^, and lymphocytes^51–53^. More recent studies have documented enhanced eosinophilic inflammation in the lung in patients with fatal COVID-19 infection^54,55^. By using flow cytometric analysis of whole blood samples obtained longitudinally during the acute and recovery phases of COVID-19 infection, Rodriguez *et al.* demonstrated transient expansion of activated CD62L^+^ eosinophils mediated by interferon-gamma (IFN-γ) and suggest that this immunomodulation may promote eosinophil trafficking to the lung following acute infection, providing a potential mechanism for COVID-19-associated eosinophilic lung pathology^56^. Albeit uncommon, case reports describing the temporal association of COVID-19 immunization and the development of eosinophilic disorders, including acute eosinophilic pneumonia and eosinophilic granulomatosis with polyangiitis^57–59^, are intriguing in this regard and merit further investigation.

Whereas eosinophils may be associated with disease pathogenesis in COVID-19 infection, pre-existing eosinophilic conditions do not appear to be a risk factor for severe illness^60,61^. In fact, some studies have suggested that type 2 inflammation may exert a protective effect by limiting viral entry through modulation of angiotensin-converting enzyme 2 (ACE2) expression at respiratory and gastrointestinal (GI) epithelial surfaces^62–66^. Consistent with this hypothesis, analysis of a large database of hospitalized patients with COVID-19 infection demonstrated improved survival in patients with a history of eosinophilic gastrointestinal disorders (EGIDs) compared with non-EGID patients matched for demographic and clinical factors^67^, and pre-existing eosinophilia was associated with a lower hospitalization rate and lower mortality in a retrospective analysis of 951 asthma patients presenting with COVID-19^68^. These findings have led to the hypothesis that eosinophilia or Th2-high inflammation (or both) may be protective in COVID-19^69^ and conversely that the use of eosinophil-depleting therapies may be harmful. Although definitive clinical studies addressing these concerns are lacking, case reports of mild disease in high-risk patients with asthma on eosinophil-depleting biologic therapy^70,71^ and data from published studies examining the association between biologic therapy and the incidence and severity of COVID-19 infections in asthmatic patients (Table 1) suggest that pharmacologic depletion of eosinophils does not increase susceptibility to or adversely affect the outcome of COVID-19 infection.

**Table 1.** Eosinophil-depleting biologics and COVID-19 in asthma.

Nature of study	Population	Sample size	Major finding(s)	Reference
Prospective — observational	Adults with severe asthma enrolled in Dutch Severe Asthma Registry (RAPSODI)	Total: 707 COVID-19+: 10 Receiving biologic therapy: 634	Incidence and severity of COVID-19 infection were increased in patients receiving biologics compared with the general Dutch population but comparable to severe asthmatics.	73
Prospective — observational	Adults with severe asthma enrolled in Severe Asthma Network in Italy (SANI)	Total: 1,504 COVID-19 suspected or confirmed: 26 Receiving biologic therapy: 65%	Biologic use was not a risk factor for infection or for more severe infection.	74
Retrospective — electronic health record analysis	Adult asthmatics	Total: 71,182 COVID-19+: 1,006 Receiving biologic therapy: 865	Despite increased asthma severity in patients receiving biologics, hospitalization and mortality were similar to those in the cohort as a whole.	75
Retrospective — electronic database and health record analysis	Non-obese adults	No asthma: 62,042 Inactive asthma: 3,890 Active asthma: 6,130	Asthma severity was positively associated with higher hospitalization risk (OR 2.89) but not with increased risk of ICU admission. Patients on Th2 biologics (n = 54) had an increased risk of hospitalization (OR 3.3).	76
Retrospective — electronic database analysis	Adult asthmatics who underwent PCR testing for COVID-19	Total: 80,602 COVID-19+: 8,242 Receiving biologic therapy: 50	Biologic use was not a risk factor for infection or for more severe infection.	77
Survey	Belgian Severe Asthma Cohort	Total: 676 COVID-19+: 14 Receiving biologic therapy: 434	Although COVID-19 infection was rare, there was no increase in incidence or severity in asthmatic patients receiving biologic therapy.	78
Survey	Adult asthmatics receiving omalizumab or mepolizumab	75	Patients who discontinued their biologic for logistical reasons during the pandemic were more likely to contract COVID-19.	79
Survey	Italian Registry of Severe Asthma	558 COVID-19+: 7 Receiving biologic therapy: 129	Biologic use was not a risk factor for infection or for more severe infection.	80
Survey	Adults with severe asthma receiving biologic therapy	Total: 473 COVID-19: 4 Receiving biologic therapy: 473	Fifteen patients underwent symptomatic COVID-19 testing, of which only four were PCR-positive. Two patients receiving omalizumab were admitted to the ICU and one died. One patient receiving benralizumab had only mild symptoms.	81

Articles (n = 1,762) identified from a PubMed search conducted on February 6, 2022, using the terms “asthma AND COVID-19” were reviewed for evidence of primary data related to biologic therapy and COVID-19 infection. The listed studies are limited to those that included at least 50 adults receiving biologic therapy. ICU, intensive care unit; OR, odds ratio; PCR, polymerase chain reaction.

## Eosinophil heterogeneity in homeostasis and immunity

In a landmark paper published in 2010, Lee *et al.* argue that the main function of eosinophils is to regulate “local immunity and remodeling/repair in both health and disease”^72^. This concept is now widely accepted, and eosinophils have subsequently been implicated in a host of homeostatic and immune processes^82^, including glucose metabolism in adipose tissue^83,84^, maintenance of long-lived plasma cells in the bone marrow and gut^85,86^, tissue repair in response to injury^87,88^ or autoimmunity^89^, and protective responses to classic type 1 pathogens, such as viruses, *Helicobacter pylori*^90^, *Mycobacterium tuberculosis*^91^, and *Leishmania major*^92^.

The dual roles of the eosinophil in producing and downregulating inflammation suggest that, like other immune cells, functional subtypes of eosinophils may exist. This hypothesis is supported by the fact that eosinophils can be detected in healthy tissues in the absence of inflammation. Although these “resident eosinophils” (rEos) are most prominent in the GI tract^93,94^, small numbers of rEos have been demonstrated by flow cytometry and immunohistochemistry in multiple sites, including the airways^95^, the thymus^96,97^, and adipose tissue^83,84^, in mice and humans. Moreover, intravital imaging and the use of reporter mice have identified rare eosinophil populations in additional tissues, including the skin and liver, and characterized their morphology and behavior in response to allergen challenges^98^. Recent work has focused on two interrelated themes: (1) characterizing the phenotype of rEos compared with that of eosinophils involved in the inflammatory response (iEos) and (2) functional differences between rEos and iEos.

Phenotypic differences between eosinophils, often with functional consequences, have been demonstrated by multiple groups (Table 2). In the murine lung, two distinct subsets of parenchymal rEos were identified based on Siglec-F^int^CD62L^+^CD101^lo^ surface expression. These cells have a ring-shaped nucleus and a regulatory gene expression profile^95^. Functionally, they inhibit dendritic cell maturation and Th2 sensitization to an allergen. In contrast, iEos in the same model were localized in peribronchial areas, had segmented nuclei, were characterized by a Siglec-F^hi^CD62L^–^CD101^hi^ surface phenotype, and exhibited a proinflammatory gene signature and Th2 effector functions^95^. Eosinophil surface expression of Gr1 and Ly6G in the murine lung in the setting of allergen challenge or viral infection (or both) also identifies two populations of eosinophils characterized by distinct granule-derived cytokine repertoires suggestive of inflammatory (IL-13, CXCL13, and IL-27) versus regulatory (CXCL10 and IL-10) functions^99^.

**Table 2.** Phenotypic classification of eosinophils.

Source of eosinophils	Species	Condition	Immunophenotype^a^	Proposed type	Functional characteristics	Reference
Lung	Mouse	Naïve and allergen challenge	Siglec-F^int^ CD125^int^ CD62L^+^ CD101^lo^	rEos	Inhibited dendritic cell maturation and Th2 sensitization to allergen	95
Lung	Mouse	Allergen challenge	Siglec-F^hi^ CD125^int^ CD62L^−^ CD101^hi^	iEos	Th2 effector functions	95
Lung	Mouse	Allergen challenge	Siglec-F^+^ Gr1^hi^Ly6G^+^ CD11c^neg^	iEos	Inflammatory mediator content: IL-13, CXCL13, and IL-27	99
Lung	Mouse	Allergen challenge	Siglec-F^+^ Gr1^neg^ CD11c^neg^	rEos	Regulatory mediator content: CXCL10, IL-10	99
Small bowel (intraepithelial)	Mouse	Pulmonary allergen challenge	Siglec-F^hi^ CD11b^hi^ CD11c^+^	rEos	Not tested	102
Small bowel (lamina propria)	Mouse	Pulmonary allergen challenge	Siglec-F^int^ CD11b^−^ CD11c^−^	iEos	Not tested	102
Lung	Mouse	Oral allergen challenge with ovalbumin	Siglec-F^int^ CD11c^int^	iEos	Increased mucus production; potentiation of response to intranasal house dust mite	102
Small bowel (lamina propria)	Mouse	Naïve WT	SSC^hi^ Siglec-F^lo^ CD11c^−/lo^ CD62L^−^	rEos	Not tested	103
Small bowel (villi)	Mouse	OVA challenge, TNF^ΔARE^	SSC^lo-int^ Siglec-F^int^ CD11c^int^ CD62L^−^	iEos	Not tested	103
Small bowel (intraepithelial)	Mouse	OVA challenge, TNF^ΔARE^	SSC^lo-int^ Siglec-F^hi^ CD11c^hi^ CD62L^−^	iEos	Not tested	103
Small bowel (transepithelial)	Mouse	OVA challenge	Siglec-F^+^ CD11c^lo^ CD11b^hi^	iEos	Not tested	103
Small bowel (intraepithelial)	Mouse	Naïve WT	Siglec-F^hi^ CD11b^hi^ CD11c^+^ MHCII^+^CD80^+^	rEos	Antigen presentation	101
Small bowel (lamina propria); blood	Mouse	Naïve WT	Siglec-F^int^ CD11b^−^ CD11c^−^ MHCII^−^CD80^−^	iEos	Not tested	101
Joint	Mouse	Allergen challenge	Siglec-F^int^ Ly6G^neg^ CD11b^+^	rEos	rEos RNA-seq profile; reduced inflammatory arthritis *in vivo*; induced alternatively activated macrophages *in vitro*	89
Lung	Mouse	Allergen challenge	Siglec-F^hi^ Ly6G^neg^ CD11b^+^	iEos	iEos RNA-seq phenotype; produce IL-5	89
Lung	Human	Normal and asthmatic	Siglec-8^+^ CD62L^+^ IL-3R^lo^	rEos	Not tested	95
Sputum	Human	Asthmatic	Siglec-8^+^ CD62L^lo^ IL-3R^hi^	iEos	Not tested	95
Blood	Human	Healthy and COVID-19- infected	CD16^lo^ CD62L^+^ CD11b^mod^	rEos	Dominant population in the absence of activation in healthy and COVID-19-infected individuals	104
Blood	Human	Healthy and COVID-19- infected	CD16^lo^ CD62L^lo^ CD11b^bright^	iEos	Increased in response to activation by formyl peptide in healthy individuals but not in patients with COVID-19 infection	104
Blood	Human	Rheumatoid arthritis in remission	CD125^int^ Siglec-8^+^ CD62L^+^	rEos	Not tested	89
Blood	Human	Healthy	Siglec-8^lo^ CCR3^+^ CD16^hi^	rEos	Inhibition of T-cell proliferation	105

^a^Eosinophils were identified by SSC^hi^ in all studies and, where reported, by CD45^+^. iEos, inflammatory eosinophils; IL, interleukin; OVA, ovalbumin; rEos, resident eosinophils; RNA-seq, RNA-sequencing, WT, wild-type.

Varied eosinophil surface phenotypes have also been described in the murine GI tract (reviewed in 100). In one study, two distinct populations of rEos in the small bowel of naïve wild-type mice could be distinguished from blood eosinophils by the increased expression of CD11b (integrin alpha-M), Siglec F, and CD11c (ICAM-1) as well as the constitutive expression of molecules involved in antigen presentation (MHC II and CD80)^101^. The functionality of these receptors was confirmed *in vivo* using a ligated intestinal loop model of luminal antigen uptake in allergen-sensitized mice. It should be noted that upregulation of MHC class II and CD80 and antigen presentation by eosinophils has also been described on bronchoalveolar lavage eosinophils from aerosol allergen-challenged mice^106^ and on human blood eosinophils exposed to various allergens and bacterial products *in vitro*^107^, suggesting that the functional distinctions between resident eosinophils (rEos) and those recruited from the blood (iEos) may not be rigid.

The fact that eosinophils recruited to the tissue can play a homeostatic, rather than an inflammatory, role in multiple settings supports this plasticity of eosinophil function. For example, eosinophils recruited to the liver have been shown to promote hepatocyte proliferation and liver regeneration following partial hepatectomy or intraperitoneal injection of carbon tetrachloride through an IL-4-dependent mechanism^108^ and to mitigate hepatic ischemic reperfusion injury in mice via IL-33-ST2-dependent production of IL-13 and suppression of neutrophil influx into the injured liver^87^. Similarly, in the heart, activated blood eosinophils recruited to the area of infarct on day 1 following experimentally induced myocardial infarction in mice secrete IL-4 and modulate the early influx of neutrophils and monocytes, resulting in improved cardiac function^88^. Finally, eotaxin-2-dependent eosinophil recruitment to the skin has been shown to maintain the anti-inflammatory function of dermal macrophages in mice with cutaneous leishmaniasis in an IL-4-dependent manner^92^.

Interesting recent work has begun to explore the role of eosinophils in crosstalk between different sites of allergic sensitization. Olbrich *et al.* observed eosinophilic infiltration within the unexposed lung and intestine following cutaneous, airway, or oral allergen challenge in mice one week following intraperitoneal sensitization to ovalbumin^102^. The two phenotypes of remote-elicited small bowel eosinophils were identical to those described previously and consistent with rEos^101^. In contrast, not only did airway eosinophils recruited after oral challenge demonstrate an activated phenotype (Siglec-F^+^CD11c^+^), but their presence enhanced local inflammatory responses to an unrelated inhaled allergen (house dust mite). This site-dependent effect was also seen using a combined asthma/arthritis model, where increases in synovial eosinophils were associated with attenuation of joint inflammation in mice sensitized and challenged with ovalbumin^89^. In this model, the joint eosinophils had phenotypic, genotypic, and functional characteristics consistent with rEos (in contrast to iEos in the lung) and were induced to proliferate *in situ* by increased systemic IL-5 produced primarily by ILC2 and CD4^+^ cells in the lung. Sensitization and challenge with house dust mite (instead of ovalbumin) caused a dominant Th17 response and exacerbation of arthritis^89^, suggesting that both the site of sensitization and the antigen are important drivers of the type of eosinophilic response. A recent study demonstrating that exposure of murine eosinophils to type 1 (IFN-γ or *Escherichia coli* or both) or type 2 (IL-4) conditions *in vitro* were sufficient to produce distinct transcriptional signatures further supports the hypothesis that the environment plays an important role in driving eosinophil heterogeneity^109^.

Although regulatory functions and phenotypic heterogeneity of eosinophils have clearly been demonstrated in humans (Table 2), the relationship between phenotype and function is much less clear. Substantial circumstantial evidence for this relationship includes a subpopulation of CD16^hi^ human eosinophils that is increased in allergic donors and after allergen provocation^110^, phenotypic differences between eosinophils in normal lung tissue and those from the sputum of individuals with allergic asthma^95^, and an increased number of eosinophils with a regulatory phenotype in the peripheral blood and joint synovium of patients with rheumatoid arthritis in remission compared with patients with active disease^89^. However, the functional heterogeneity of human eosinophils has been examined directly in only a few studies to date. In one such study, Lingblom *et al.* demonstrated greater suppression of T-cell proliferation by CD16^hi^ eosinophils from healthy donors compared with CD16^lo^ eosinophils from the same donors^105^. This suppression was at least partly dependent on galectin-10 (Gal-10) released in extracellular DNA traps when eosinophils come into contact with activated T cells^111^. An area of ongoing controversy, especially in humans, is whether there are developmentally distinct populations of eosinophils that maintain their phenotype and function irrespective of their environment or whether the observed heterogeneity is entirely due to plasticity in response to external cues. This has important implications in the context of the ever-increasing number of targeted therapeutic agents that deplete eosinophils or alter their cytokine milieu (or do both).

## Lessons learned from eosinophil-targeted therapies

The advent of eosinophil-targeted therapies not only has revolutionized the landscape of treatment for eosinophilic disorders by providing less toxic and more effective therapeutic options for patients with common and rare eosinophil-associated disorders but has provided a unique opportunity to enhance our understanding of the basic biology of eosinophils in humans and their role in homeostasis and disease. Whereas a comprehensive discussion of the many novel therapies currently available and in clinical development for eosinophil-associated diseases is beyond the scope of this article and can be found in numerous reviews^112–114^, a general discussion of the differences between eosinophil-targeted agents will be provided, and specific examples will be utilized to illustrate the concepts outlined above.

### The armamentarium of eosinophil-targeted therapies

**Figure 1.**
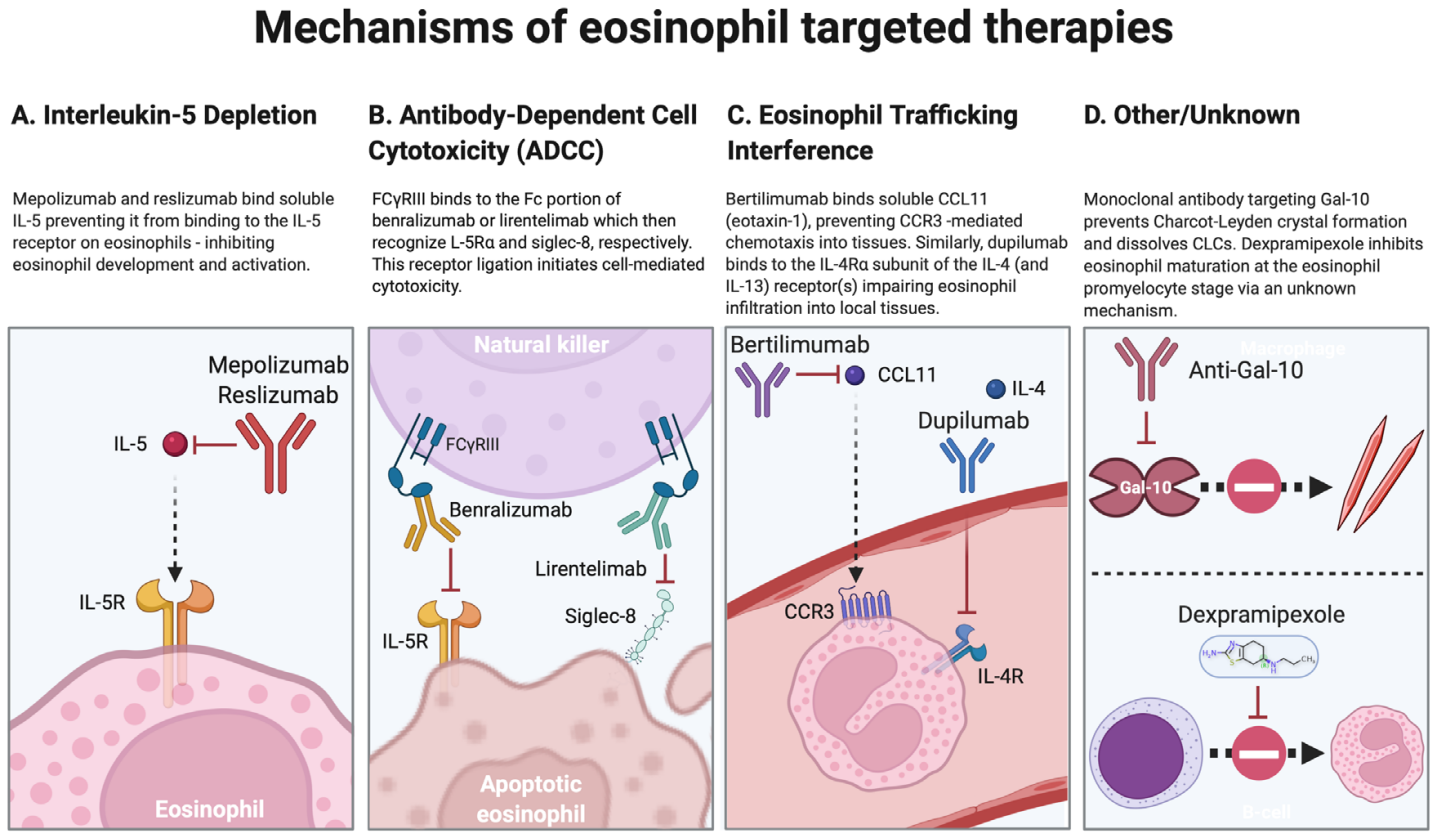
Mechanisms of eosinophil-targeted therapies. Categorization of eosinophil-targeted therapies by broad mechanisms of action: (**A**) depletion of interleukin 5 (IL-5) (mepolizumab and reslizumab), (**B**) antibody-dependent cytotoxicity (benralizumab and lirentelimab), (**C**) interference with eosinophil trafficking to tissues (e.g., dupilumab and bertilimumab), and (**D**) other or unknown. Select examples, including clinically available and investigational drugs, are shown. Created with BioRender.com. CLC, Charcot-Leyden crystal; Gal-10, galectin-10.

Eosinophil-targeted therapies can be broadly classified by their mechanisms of eosinophil reduction: (1) depletion of IL-5 (mepolizumab and reslizumab), (2) antibody-dependent cytotoxicity (benralizumab and lirentelimab), (3) interference with eosinophil trafficking to tissues (e.g., dupilumab and bertilimumab), and (4) other or unknown (Figure 1). The varied agents also differ in their impact on eosinophil levels in different compartments (bone marrow and blood or tissue or both) and the effects on cells other than eosinophils. These differences have influenced the diseases chosen for clinical development and approved indications (Table 3).

**Table 3.** Selected eosinophil-targeting biologics approved or in development for eosinophil-associated diseases.

Medication	Molecular target	Mechanism of action	Approved indication(s) (year approved)	Indication(s) under investigation
Mepolizumab	IL-5	Prevents IL-5 from interacting with the IL-5 receptor	Severe asthma (2015) EGPA (2017) HES (2020) CRSwNP (2021)	Eosinophilic fasciitis, EAE, COPD, EoE
Depemokimab	IL-5	Extended half-life mAb that prevents IL-5 interaction with the IL-5 receptor		Asthma
Reslizumab	IL-5	Prevents IL-5 from interacting with the IL-5 receptor	Severe asthma (2016)	HES, EoE, EGPA
Benralizumab	IL-5Rα	ADCC	Severe asthma (2017)	HES, EoE, EoG, EGPA, atopic dermatitis, CRSwNP, bullous pemphigoid
Dupilumab	IL-4Rα	Inhibition of eosinophil migration into affected tissues	Atopic dermatitis (2017) Severe asthma (2018) CRSwNP (2019) EoE (2022)	EoG, EoN, pruritis, keloids, food allergy, CSU, bullous pemphigoid, AERD, NSCLC
Lirentelimab	Siglec-8	ADCC		EoE, EoG, EoN, CSU, severe allergic conjunctivitis, indolent systemic mastocytosis
Bertilimumab	CCL11	CCL11 blockade, inhibiting eosinophil migration		Bullous pemphigoid
Tezepelumab-ekko	TSLP	Prevents TSLP from interacting with TSLP receptor, reducing blood and tissue eosinophils	Severe asthma (2021)	EoE, COPD, CSU, CRSwNP

ADCC, antibody-dependent cell-mediated cytotoxicity; AERD, aspirin-exacerbated respiratory disease; COPD, chronic obstructive pulmonary disease; CRSwNP, chronic rhinosinusitis with nasal polyps; CSU, chronic spontaneous urticatia; EAE, episodic angioedema with eosinophilia; EGPA, eosinophilic granulomatosis with polyangiitis; EoE, eosinophilic esophagitis; EoG, eosinophilic gastritis; EoN, eosinophilic enteritis; HES, hypereosinophilic syndrome; IL, interleukin; mAb, monoclonal antibody; NSCLC, non-small cell lung cancer; TSLP, thymic stromal lymphopoietin.

### Eosinophils in disease pathogenesis

The earliest example of the utility of eosinophil-targeted therapy in understanding disease pathogenesis in humans comes from the initial trials of mepolizumab and reslizumab in asthma, which failed to show significant improvement in clinical outcome measures^115,116^, leading to concern that eosinophils were unimportant in asthma pathogenesis. Instead, these initial stumbling blocks led to the recognition of asthma endotypes that continue to help define the pathogenesis and treatment of severe asthma^117^. More recently, it has been suggested that variability exists even within the endotype of eosinophilic asthma, with implications for the selection of targeted therapies^118^. In a recent retrospective analysis of 3,531 patients with severe asthma, 384 (11%) switched biologics once and 45 switched twice, consistent with this hypothesis^119^. Similar findings have been reported in a multicenter retrospective study of patients with *PDGFRA*-negative hypereosinophilic syndrome (HES), which described successful switching in the subset of patients who did not respond to the initial biologic tried^120^.

The apparent disconnect between eosinophil reduction and clinical outcomes also reminds us that multiple cell types and pathways may play a role in eosinophil-associated disorders. This has been particularly evident in EGIDs, where clinical trials with mepolizumab^121,122^, reslizumab^123^, and benralizumab (ClinicalTrials.gov Identifier: NCT03473977) have not met clinical endpoints despite substantial or, in the case of benralizumab, near complete reduction of blood and tissue eosinophilia. An analysis of GI pathology specimens from a phase 2 clinical trial of benralizumab in HES demonstrated persistent epithelial changes in the stomach and esophagus despite eosinophil depletion, consistent with a role for factors other than eosinophils in disease pathogenesis in EGIDs^124^. The promising clinical outcome results from phase 2 trials of lirentelimab, a mAb to Siglec-8 that both depletes eosinophils and inhibits mast cell degranulation^125^, and dupilumab, a mAb that targets IL-4 receptor alpha reducing tissue eosinophilia and blocking IL-4/IL-13 signaling^126^, also suggest that targeting multiple pathways may be more effective. Unfortunately, these findings have not been replicated in phase 3 studies to date (ClinicalTrials.gov Identifiers: NCT04322604 and NCT04322708). More studies using targeted therapies are needed to disentangle the role of eosinophils in the pathogenesis of EGIDs and other eosinophil-associated diseases. These should include head-to-head studies, such as the ongoing trial comparing mepolizumab with benralizumab for the treatment of eosinophilic granulomatosis with polyangiitis (ClinicalTrials.gov Identifier: NCT04157348), to directly assess the effects of differing degrees and methods of eosinophil depletion in the different disorders and patient populations.

### Eosinophils and homeostasis

Despite convincing evidence supporting a homeostatic role for eosinophils, long-term safety studies using eosinophil-depleting agents have failed to demonstrate major negative consequences after 3 to 11 years of therapy^127–132^. That said, the relatively few human studies that have looked directly at more subtle changes in immune responses have not replicated the data from murine models (reviewed in 133. For example, whereas mice exhibited decreased recall responses to immunization with a T cell-dependent antigen^134^, the responses to tetanus^135^ and influenza^136^ vaccination were unaffected by benralizumab. Whereas the lack of effect could be due to the fact that the mice were devoid of eosinophils from the time of conception, data from non-human primates and a single case of a human baby born to a mother receiving benralizumab suggest that the timing of eosinophil depletion is not the explanation^137^. Although a recent study by Andreev *et al.* described a cohort of eight patients with rheumatoid arthritis, six of whom flared following initiation of treatment with mepolizumab, suggesting that IL-5 blockade may impair or reduce the function of regulatory eosinophils, no information is provided regarding tapering of concomitant therapies or other confounding variables during this time^89^. Eosinophil-deficient mice have normal fertility^138,139^, suggesting that eosinophils are not essential for normal reproduction. Although published data in humans are limited to a handful of case reports, data are currently being collected through a registry (https://mothertobaby.org) and should provide a definitive answer to the question of whether eosinophil depletion is safe in pregnancy. Other potential consequences of prolonged eosinopenia, including effects on susceptibility or severity of helminth infection, have not been formally studied in humans.

Even if (as current data suggest) eosinophil depletion is safe in humans, this may reflect the fact that essential eosinophil functions are only part of a network of responses. In this regard, the potential for biologics that target multiple cells or pathways and/or the concomitant use of multiple biologics to unmask adverse consequences remains a theoretical concern. Although studies using lirentelimab and case reports describing dual biologic therapy have not supported this hypothesis to date, long-term data are lacking. Similarly, there is little evidence that dupilumab, which decreases tissue eosinophilia and blocks IL-4 and IL-13 signaling, adversely affects tissue repair, despite a growing body of evidence pointing to the importance of eosinophil secretion of IL-4 in this process^109^.

### Novel targets

Novel targets are of particular interest in the management of eosinophilic disorders and may provide alternatives for individuals or subpopulations who do not respond to existing therapeutics and may help define the roles of novel molecules and pathways in eosinophil biology. This is illustrated by recent work from Persson *et al.* demonstrating that Gal-10 protein, the major component of Charcot-Leyden crystals, promotes goblet cell metaplasia and bronchial hyperreactivity in a mouse model of asthma. Importantly, antibodies against Gal-10 were found to dissolve Charcot-Leyden crystals and ameliorate type 2 airway inflammation^140^ (Figure 1D), providing a potentially novel approach to the treatment of eosinophilic inflammation, particularly in the context of eosinophilic asthma. The small molecule dexpramipexole, an orally bioavailable R-enantiomer of the dopamine agonist pramipexole, provides a second example. Incidentally found to cause eosinopenia during a phase 3 trial in patients with amyotrophic lateral sclerosis^141^, dexpramipexole was subsequently shown to deplete blood and tissue eosinophils in patients with chronic rhinosinusitis with nasal polyps^142^ and HES^143^. Most recently, dexpramipexole has shown efficacy in reducing eosinophilia and improving lung function in a phase 2 trial in asthmatics (ClinicalTrials.gov Identifier: NCT04046939). Although the mechanism of action is unknown, bone marrow sampling during the study in HES was consistent with induction of maturational arrest of eosinophils at the promyelocyte stage.

## Conclusions

Recent advances in our understanding of eosinophil biology have continued to highlight the multifunctional and complex nature of this mysterious cell. Currently available drugs that target eosinophils, as well as new additions to the therapeutic arsenal, provide not only hope for patients with eosinophilic disorders but a unique opportunity to illuminate the role of eosinophils in different contexts. Careful attention to trial design and associated mechanistic studies is essential in this regard.
